# OP49 Are Propensity-Score-Based Adjusted Indirect Comparisons Feasible For All European Joint Clinical Assessments Based On Non-Randomized Data?

**DOI:** 10.1017/S0266462324001119

**Published:** 2025-01-07

**Authors:** Robert Krüger, Chiara Cantoni, Anke Van Engen

## Abstract

**Introduction:**

The EU HTA member state coordination group has finalized methodological guidance on indirect comparisons that states that propensity score (PS) methods should generally be used for indirect comparisons of non-randomized data in joint clinical assessments (JCAs). Half of new oncology approvals by the European Medicines Agency (EMA) between 2020 and 2023 were based on non-randomized data. This study aimed to identify how many of these were able to submit PS-based comparisons.

**Methods:**

Using IQVIA’s Market Access Insights (MAI) database of HTAs and regulatory approvals, we characterized evidence packages submitted to EMA and HTA agencies of EU member states according to the use of PS-based comparisons and access to individual patient data (IPD) from comparator studies.

**Results:**

Of the 56 oncology approvals between 2020 and 2023, 30 (54%) were based on non-randomized data, of which 23 (23%) submitted PS-based indirect comparisons to EMA (15 therapies) or to HTA agencies (23 therapies). Electronic health record (EHR) or chart reviews were the most common source of comparative RWE, but agencies only took this evidence into account in fewer than half of HTAs where it was available. Use of PS-based methods also did not lead to more positive HTA outcomes than the alternative unanchored matching-adjusted indirect comparisons (MAICs) to aggregated data.

**Conclusions:**

The prevalence of oncology approvals based on single-arm trials is expected to be a key challenge to the success of JCA. Unanchored comparisons will be required, but IPD was not necessarily shown to reduce uncertainty in HTAs analyzed in this research, and in about half of cases, comparisons to aggregate data were preferred due to applicability and heterogeneity concerns. Thus, the source of comparator data appears more relevant than the comparison method in HTAs, which contrasts with the available EU HTA coordination group guidance that focuses mainly on methodological aspects.

